# Endocrine-driven inflammatory–epigenetic adaptation as a proposed axis of radioresistance in endocrine-exposed prostate cancer: a translational hypothesis

**DOI:** 10.3389/fendo.2026.1851378

**Published:** 2026-06-23

**Authors:** Miloš Grujić, Marija Živković Radojević, Tinatin Alaverdashvili, Imdat Eroglu, Katarina Krasić, Katarina Janković, Neda Milosavljević

**Affiliations:** 1 University of Kragujevac, Serbia, Faculty of Medical Sciences, Kragujevac, Serbia; 2 Clinic of Radiation Oncology, University Clinical Center Kragujevac, Kragujevac, Serbia; 3 University of Kragujevac, Serbia, Faculty of Medical Sciences, Department of Clinical Oncology, Kragujevac, Serbia; 4 Radiation Oncology Department, Todua Clinic, Tbilisi, Georgia; 5 Gazi University School of Medicine, Department of Medical Oncology, Ankara, Türkiye

**Keywords:** androgen deprivation therapy, androgen receptor, epigenetic remodeling, IL-6/STAT3, lineage plasticity, prostate cancer, radioresistance, radiotherapy

## Abstract

Radiotherapy response in prostate cancer is commonly interpreted through partly separate frameworks centered on androgen receptor (AR) signaling, DNA damage response (DDR), inflammatory adaptation, and lineage plasticity. Here, we propose a translational hypothesis particularly relevant to endocrine-exposed disease in settings of biologically meaningful endocrine exposure before or during radiotherapy, especially high-risk localized, locally advanced, and selected salvage settings: the radiosensitizing effect of AR suppression may be temporally dynamic rather than fixed. In a subset of tumors, sustained endocrine pressure may initially impair AR-dependent DDR signaling but subsequently promote adaptive inflammatory signaling and epigenetic remodeling that attenuate this benefit and favor relative radioresistance. We propose a sequence-level model in which endocrine therapy acts as the initiating biologic stressor, inflammatory signaling functions as the adaptive amplifier, and epigenetic remodeling stabilizes a more durable treatment-tolerant state that becomes functionally revealed in the radiotherapy setting. While several bilateral links within this framework are supported by existing literature, the full temporally ordered cascade should be regarded as inferential and hypothesis-generating. The novelty of this framework lies not in proposing a new pathway, but in reframing endocrine-associated radiosensitization as a dynamic biologic state that may diverge over time across apparently similar treatment paradigms. This model yields clear translational predictions: endocrine-exposed tumors should differ in their propensity to develop adaptive inflammatory activation, inflammatory-high states should show attenuated radiosensitization and more repair-supportive features, and serial biomarker profiling across endocrine exposure and radiotherapy should outperform baseline-only assessment for identifying emerging resistance.

## Introduction

Prostate cancer remains a major global health burden and ranks among the most frequently diagnosed cancers in men worldwide (1). Despite major therapeutic advances, treatment resistance continues to drive progression and cancer-related mortality (2). Although androgen deprivation therapy (ADT) has long been a cornerstone of management, endocrine therapy in this manuscript refers primarily to ADT together with androgen receptor (AR) pathway inhibition more broadly. Most advanced tumors eventually adapt through restoration, bypass, or recontextualization of androgen receptor (AR) signaling, often accompanied by broader transcriptional and phenotypic plasticity (2). Recent work further suggests that this adaptation is not purely genomic, but also depends on dynamic interactions between endocrine signaling, chromatin state, and the tumor microenvironment (2, 3).

Radiotherapy is a central treatment modality across localized, locally advanced, and selected oligometastatic states of prostate cancer, yet radioresistance remains a clinically important barrier to durable disease control (4). Mechanistically, prostate cancer radioresistance has been linked to enhanced DNA damage response (DDR) capacity, altered cell-state plasticity, and adaptive survival signaling (4, 5). Among these mechanisms, AR signaling has a direct relationship with DNA repair biology. Foundational studies demonstrated that AR signaling regulates transcriptional programs involved in DNA repair, while subsequent work showed that castration or potent antiandrogen treatment can impair double-strand break repair and radiosensitize prostate cancer tissue and models (6–8). Together, these observations support AR–DDR coupling as a major determinant of response to genotoxic therapy, including radiotherapy (5).

At the same time, endocrine pressure, here referring primarily to ADT and AR pathway inhibition, does not act in isolation. A growing body of literature implicates inflammatory signaling, particularly the IL-6/STAT3 axis, in prostate cancer progression, castration resistance, and treatment adaptation (9, 10). IL-6 signaling has also been directly linked to prostate cancer radioresistance through upregulation of DDR-associated molecules such as ATM, ATR, BRCA1, and BRCA2, providing a biologically plausible bridge between endocrine stress, inflammatory adaptation, and diminished radiation response (9).

Epigenetic remodeling provides a second major layer of adaptive stability. The AR is not merely a hormone-responsive transcription factor but also an organizer of chromatin structure and epigenetic regulation (3, 11). In parallel, newer work on transcriptional and epigenetic reprogramming has placed lineage plasticity and resistant cell states at the center of disease evolution under therapeutic pressure (2). More broadly, epigenetic mechanisms are increasingly recognized as mediators of therapeutic resistance across cancers because they can stabilize stress-adapted transcriptional programs and permit durable shifts in cell identity (12). These observations raise the possibility that inflammatory signals emerging under endocrine pressure may become fixed through chromatin remodeling, enhancer rewiring, and DNA methylation changes, thereby supporting persistent resistance phenotypes.

Radiotherapy itself is also not biologically neutral. Beyond inducing DNA damage, radiotherapy can reshape immune signaling and systemic inflammatory responses (13). Clinical proteomic and metabolomic data from patients treated for prostate cancer have shown that radiotherapy induces measurable innate immune responses (13), while broader reviews describe radiotherapy as an immunomodulatory intervention capable of altering cytokine production, antigen presentation, and tumor–host interactions (14). Taken together, these findings suggest that radiotherapy may not only be affected by pre-existing endocrine and epigenetic states, but may also participate in reinforcing the adaptive programs that limit its own efficacy.

Despite converging evidence linking AR signaling, inflammatory adaptation, epigenetic plasticity, and radiotherapy response in prostate cancer, these processes are still usually discussed as partially overlapping but conceptually separate domains. We propose a more specific interpretation: in endocrine-exposed prostate cancer, particularly when AR pathway suppression is sustained long enough to precede or accompany radiotherapy, endocrine-associated radiosensitization may be temporally dynamic rather than biologically fixed. In this model, endocrine therapy first perturbs AR-dependent DDR biology, but in a subset of tumors prolonged endocrine pressure may subsequently promote inflammatory amplification and epigenetic stabilization of adaptive states that attenuate that initial benefit. The novelty of the present article therefore lies not in any single pathway, but in the proposal of a sequence-level adaptive model that explains why tumors exposed to broadly similar endocrine–radiotherapy paradigms may diverge in radiotherapy responsiveness over time. The strongest evidence currently supports the bilateral mechanistic links within this framework, whereas the full temporally ordered sequence remains inferential and hypothesis-generating. Accordingly, the present article does not claim that the complete endocrine-to-inflammatory-to-epigenetic cascade has been demonstrated as a unified clinical mechanism, but rather proposes this temporal integration as a testable translational model. We present this model not as a replacement for established determinants of radioresponse, but as an additional adaptive axis most likely to be clinically relevant in treatment settings that allow biologically meaningful endocrine exposure before or during radiotherapy.

## Framework rationale

**Figure 1:**
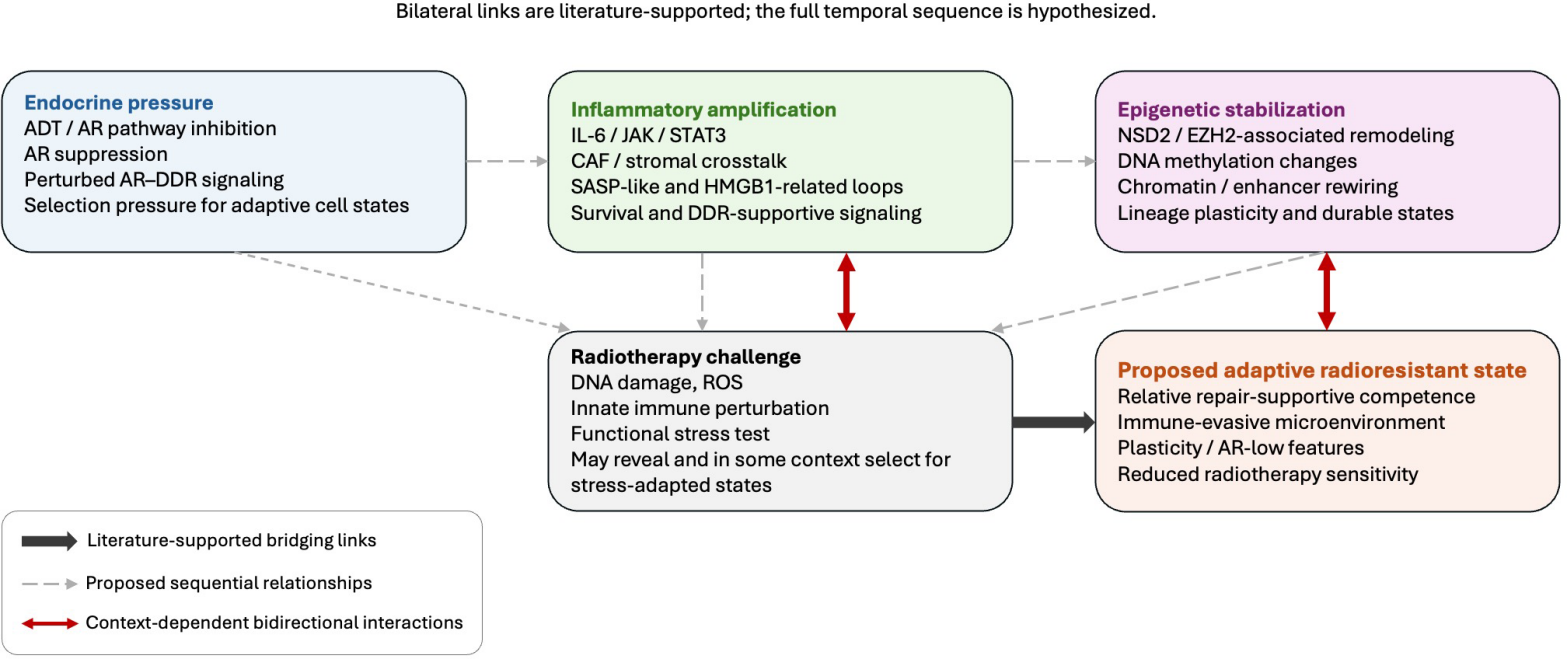
Proposed hypothesis model of endocrine-driven inflammatory–epigenetic adaptation as an axis of relative radioresistance in endocrine-exposed prostate cancer. The figure integrates literature-supported bilateral mechanistic links into a proposed temporal framework in which endocrine pressure induced by androgen deprivation therapy (ADT) and androgen receptor (AR) pathway inhibition may initiate adaptive tumor reprogramming through perturbed AR-dependent DNA damage response (DDR) signaling and selection pressure for treatment-tolerant cell states. Within this hypothesis, inflammatory amplification—particularly through IL-6/JAK/STAT3-centered signaling, stromal crosstalk, senescence-associated secretory phenotype (SASP)-like programs, and related HMGB1-associated loops—may promote survival and DDR-supportive adaptation. These states may then become more biologically durable through epigenetic remodeling, including NSD2/EZH2-associated chromatin regulation, DNA methylation changes, enhancer rewiring, and lineage plasticity. Radiotherapy is shown as the clinically revealing challenge that may expose and, in some contexts, select for stress-adapted states through DNA damage, reactive oxygen species (ROS), and immune perturbation. Black solid arrows denote literature-supported bridging links; gray dashed arrows denote hypothesized sequential relationships within the proposed model rather than established causal progression; double-headed red arrows indicate context-dependent bidirectional interactions. The bilateral links are literature-supported, whereas the full temporally ordered sequence remains hypothesis-generating. ADT, androgen deprivation therapy; AR, androgen receptor; CAF, cancer-associated fibroblast; DDR, DNA damage response; EZH2, enhancer of zeste homolog 2; HMGB1, high mobility group box 1; IL-6, interleukin-6; JAK/STAT3, Janus kinase/signal transducer and activator of transcription 3; ROS, reactive oxygen species; SASP, senescence-associated secretory phenotype.

This Hypothesis and Theory article develops a mechanism-oriented translational model linking four ordered components of treatment adaptation in endocrine-exposed prostate cancer: AR–DDR perturbation as the initiating radiosensitizing event, IL-6/STAT3-centered inflammatory signaling as the adaptive amplifier, epigenetic remodeling as the stabilizer of durable treatment-tolerant states, and radiotherapy as the clinically revealing challenge. These biologic layers and their translational relevance are summarized in **Table 1**, and the proposed sequence-level model is illustrated in **Figure 1**. Several bilateral relationships within this framework are supported by prior literature; however, their integration into a single temporally ordered adaptive sequence remains a proposed and testable hypothesis rather than an established mechanism. The aim is disciplined integration rather than exhaustive review.

**Table 1:** Conceptual framework of endocrine-driven inflammatory–epigenetic adaptation as a proposed mechanism of prostate cancer radioresistance.

Biologic layer	Key mechanism	Key mediators/processes	Translational relevance
Endocrine stress	AR suppression perturbs DDR signaling and imposes selection pressure favoring adaptive cell states	AR, DNA repair genes, DSB repair, AR–DDR coupling	The timing, intensity, and duration of endocrine exposure may influence initial radiosensitization and subsequent adaptive remodeling
Inflammatory amplification	Stress-associated inflammatory signaling promotes survival, stromal reinforcement, and DDR-supportive adaptation	IL-6, JAK/STAT3, CAF/stromal crosstalk, SASP-like signaling, HMGB1-related loops	Tumors with persistent inflammatory activation may be more likely to develop repair-supportive, less radiation-responsive states
Epigenetic stabilization	Chromatin remodeling and methylation changes stabilize therapy-tolerant and lineage-plastic cell states	NSD2, EZH2, DNA methylation, chromatin/enhancer rewiring	Adaptive states may become more durable, biologically consequential, and potentially targetable
Radiotherapy context	RT functionally reveals, interacts with, and in some contexts may select for pre-existing stress-adapted programs	DNA damage, ROS, innate immune signaling, selection of tolerant states	Serial biomarker studies may help identify endocrine-aware mechanisms and trajectories of RT resistance

The table summarizes the principal biologic layers of the proposed model, including endocrine stress, inflammatory amplification, epigenetic stabilization, and the radiotherapy context in which these adaptive programs may be functionally revealed or reinforced. For each layer, the table outlines the central mechanism, representative mediators or processes, and the main translational relevance, highlighting how endocrine exposure, inflammatory signaling, chromatin plasticity, and treatment context may collectively shape radiotherapy response. AR, androgen receptor; CAF, cancer-associated fibroblast; DDR, DNA damage response; DSB, double-strand break; EZH2, enhancer of zeste homolog 2; HMGB1, high mobility group box 1; IL-6, interleukin-6; JAK/STAT3, Janus kinase/signal transducer and activator of transcription 3; ROS, reactive oxygen species; RT, radiotherapy; SASP, senescence-associated secretory phenotype.

### Endocrine stress as the initiating event

Endocrine therapy in prostate cancer should be viewed not only as a tumor-suppressive intervention, but as a biologic perturbation with two temporally distinct consequences. The first, and best established, is radiosensitization through disruption of AR-dependent DDR signaling. AR regulates transcriptional networks involved in DNA repair, and castration or potent AR pathway inhibition can impair double-strand break repair and increase radiation sensitivity in prostate cancer tissue and models (6–8). This AR–DDR relationship provides the mechanistic starting point of the present framework.

The second consequence is more inferential but clinically important: under sustained AR pathway pressure, endocrine exposure may also impose selection for adaptive states that are no longer fully explained by the acute AR–DDR effect alone. Contemporary models of prostate cancer progression increasingly suggest that resistance emerges not only through restoration or bypass of AR signaling, but through broader transcriptional and phenotypic diversification, including lineage-plastic and treatment-tolerant states (2, 15–17). This broader framework is reinforced by recent work positioning transcriptional and epigenetic reprogramming as a central organizing principle of prostate cancer adaptation under therapeutic pressure (2). In the present hypothesis, these longer-term adaptations are what create the possibility that initial endocrine-associated radiosensitization may not remain biologically stable in all tumors.

Emerging experimental data support this broader interpretation. ADT has been shown to promote aggressive alternative phenotypes through HGF/MET- and Wnt-associated reprogramming, while recent work identified NSD2 as a critical driver of lineage plasticity, neuroendocrine-like transition, and resistance to AR inhibition in prostate cancer (16, 18). These findings suggest that endocrine pressure may actively remodel the biologic landscape from which resistance emerges, rather than merely selecting for a fixed pre-existing clone. In that sense, endocrine therapy may create the conditions under which adaptive inflammatory signaling and epigenetic stabilization become biologically consequential.

Taken together, these data support a broader interpretation of endocrine therapy in prostate cancer: beyond its acute radiosensitizing effect through AR–DDR disruption, sustained endocrine pressure may also create the biologic conditions for adaptive state transitions (2, 5–8, 15–18). In this framework, endocrine exposure is therefore not only a therapeutic intervention, but the proposed initiating context in which later resistance-promoting programs may emerge.

### Inflammatory signaling as the adaptive amplifier

If endocrine therapy initiates the adaptive trajectory, IL-6/STAT3-centered inflammatory signaling is the most plausible intermediate program through which that trajectory is amplified. Among inflammatory pathways implicated in prostate cancer, IL-6/STAT3 has the strongest combined relevance to castration resistance, microenvironmental adaptation, survival signaling, and radioresistance-associated DDR support (10, 19). In the present model, IL-6/STAT3 is therefore used as the principal inflammatory exemplar rather than the exclusive driver of adaptation. Importantly, IL-6 is not produced exclusively by tumor cells. Stromal and immune elements of the tumor microenvironment, including cancer-associated fibroblasts (CAF) and macrophage-associated signaling networks, can reinforce IL-6/STAT3 activity under treatment pressure, thereby extending adaptation beyond the malignant epithelial compartment (19).

At the cellular level, STAT3 activation promotes several processes relevant to resistance, including survival signaling, apoptosis evasion, metabolic rewiring, invasion, stem-like behavior, and immune-shaping effects (10). This makes IL-6/STAT3 more than a bystander inflammatory pathway; it represents a plausible intermediate program through which endocrine perturbation may be translated into persistent biologic fitness. In the context of the present hypothesis, this is especially important because it provides a mechanism by which endocrine-treated tumors may acquire compensatory signaling states even when canonical AR-dependent programs are initially suppressed.

The relevance of this pathway to radiotherapy response is particularly compelling. IL-6 signaling has been shown to contribute directly to prostate cancer radioresistance through upregulation of DDR-associated molecules including ATM, ATR, BRCA1, and BRCA2, with STAT3 functioning as a critical transcriptional mediator (9). This observation is central to our model because it supports the plausibility that inflammatory adaptation may help reconstruct repair-supportive competence after endocrine perturbation. In other words, endocrine therapy may transiently weaken AR-dependent repair biology, but a subsequent inflammatory program could partially offset that vulnerability by promoting alternative survival and repair-supportive signaling.

Inflammatory adaptation in prostate cancer likely extends beyond IL-6 alone. Chronic treatment exposure may generate a broader stress-associated inflammatory milieu that includes senescence-associated secretory phenotype (SASP)-like programs, cytokine-mediated stromal reinforcement, and damage-associated molecular signaling capable of reshaping neighboring tumor and immune cells (20, 21). These broader inflammatory landscapes are unlikely to be identical across all tumors, yet they support the general principle that treatment adaptation is not purely cell autonomous and may be sustained by reciprocal tumor–microenvironment feedback under endocrine pressure.

A further strength of the IL-6/STAT3-centered model is its capacity to integrate with other resistance-promoting loops. Experimental work in prostate cancer has identified HMGB1–IL-6–STAT3-associated feedback signaling linked to ADT resistance and tumor–microenvironment crosstalk, reinforcing the plausibility that once inflammatory signaling is activated, it may become self-reinforcing through reciprocal communication between tumor cells and host compartments (22). Although not all such loops have been specifically validated in radiotherapy models, they strengthen the plausibility that inflammatory signaling may act as a biologically meaningful amplifier between endocrine stress and more durable resistant states.

Taken together, these observations support inflammatory signaling, particularly IL-6/STAT3-centered networks, as a plausible adaptive amplifier in endocrine-exposed prostate cancer; however, its placement as the intermediate step within a full temporal cascade remains part of the present hypothesis. Rather than merely accompanying resistance, this layer may deepen treatment adaptation by linking endocrine perturbation to survival signaling, microenvironmental reinforcement, and DDR-supportive competence (9, 10, 19).

### Epigenetic remodeling as the stabilizer of resistance

If inflammatory adaptation functions as the amplifier, epigenetic remodeling provides the most plausible mechanism by which that adaptive state becomes biologically durable. The relevance of epigenetic remodeling in the present model is therefore not simply that it accompanies endocrine resistance, but that it may stabilize treatment-tolerant, lineage-flexible, and relatively radioresistant states after inflammatory rewiring has emerged, consistent with recent models of transcriptional and epigenetic reprogramming in prostate cancer progression and therapy resistance (2, 23). This is particularly relevant under sustained endocrine pressure, where tumor cells must either restore canonical AR signaling or access alternative lineage programs that permit survival despite continued hormonal suppression (2, 3, 23).

The AR itself is deeply embedded in epigenetic regulation. Beyond its classical role as a hormone-responsive transcription factor, AR functions within chromatin-remodeling networks and cooperates with pioneer factors, histone modifiers, and transcriptional co-regulators to shape enhancer activity and transcriptional output (3, 11). This implies that endocrine perturbation is not simply altering signaling intensity; it may also reshape the epigenetic architecture through which signaling is interpreted. In that sense, epigenetic remodeling may preserve not only altered receptor dependence and lineage flexibility, but also broader stress-response competence, including transcriptional states that influence how tumor cells interpret and survive subsequent radiation-induced damage. In the context of the present framework, that distinction is important because it provides a plausible route by which transient therapeutic stress may be converted into a more persistent biologic state.

A major consequence of such remodeling is lineage plasticity. Contemporary literature increasingly positions lineage plasticity at the center of resistance to AR-targeted therapies, particularly in the evolution toward AR-indifferent and neuroendocrine-like states (2, 16, 23). Experimental studies further suggest that this process is not merely associative but mechanistically driven by defined epigenetic regulators (2). Recent work has sharpened this concept by showing that NSD2 is not simply associated with resistant phenotypes, but can function as a critical driver of lineage plasticity and resistance to AR inhibition, supporting the idea that epigenetic machinery can actively stabilize adaptive cell-state transitions under endocrine pressure (16). LKB1 inactivation has also been linked to epigenetic remodeling, global hypomethylation, and AR-independent state transitions (24). More recent work also indicates that coordinated crosstalk between EZH2 and DNA methylation machinery may stabilize lineage-divergent phenotypes, reinforcing the concept that resistant state transitions are maintained by interacting epigenetic systems rather than isolated modifiers (25). Much of the strongest mechanistic evidence for these epigenetic transitions comes from advanced or treatment-refractory disease states, where lineage plasticity is most overt; in the present framework, these data are used not to claim equivalence with localized disease, but to support biologic plausibility that earlier endocrine-exposed tumors may variably access partial versions of the same adaptive programs.

DNA methylation changes are also relevant to this model. Prostate cancer is characterized by widespread epigenetic dysregulation, including global hypomethylation and locus-specific hypermethylation, and emerging evidence suggests that such changes may contribute not only to tumor initiation and progression but also to adaptive treatment responses (26, 27). In particular, methylation-associated suppression of AR expression provides one plausible route by which endocrine-exposed tumors could evolve toward AR-low, treatment-refractory phenotypes (28). Within the present framework, these observations support the idea that endocrine and inflammatory adaptation may eventually be fixed as altered receptor dependence and more durable resistant states.

Histone modifiers provide an additional stabilizing layer. Regulators such as NSD2 and EZH2 are especially relevant because they lie at the intersection of chromatin remodeling, lineage plasticity, and resistance to endocrine therapy (3, 12, 16, 17, 29). More broadly, epigenetic enzymes can stabilize stress-adapted transcriptional programs and suppress re-differentiation, thereby allowing transient environmental or therapeutic pressures to produce durable biologic consequences (12, 17, 29). This stabilizing function is central to the present model: if endocrine stress and inflammatory signaling generate adaptive cell states, epigenetic remodeling is the most plausible mechanism by which those states become preserved across time and treatment.

Epigenetic context is also directly relevant to radiotherapy. By influencing chromatin accessibility, transcriptional state, and DNA repair pathway utilization, epigenetic remodeling can shape cellular responses to radiation-induced damage (30, 31). In prostate cancer, emerging discussions of radioresistance increasingly place cell-state plasticity and chromatin regulation alongside classical DDR mechanisms (30, 31). Thus, in the present framework, epigenetic remodeling has a dual significance: it may stabilize endocrine- and inflammation-induced adaptive states, and it may simultaneously alter the biologic context in which radiotherapy is encountered, thereby contributing to a more durable, relatively radioresistant phenotype.

Taken together, current evidence supports epigenetic remodeling as the most plausible mechanism by which adaptive treatment-associated states become persistent, heritable across cell divisions, and increasingly difficult to reverse with recent models of prostate cancer progression emphasizing that transcriptional and epigenetic reprogramming are central to the emergence and maintenance of therapy-tolerant and lineage-plastic states (2, 16, 23–25). In the present model, its specific sequential positioning after inflammatory amplification remains inferential.

### Radiotherapy as the clinically revealing context

Radiotherapy is not merely a local cytotoxic intervention superimposed on endocrine-treated disease; it is a biologically active stressor that intersects directly with AR signaling, DDR capacity, inflammatory signaling, and cell-state plasticity (5, 30, 31). This makes radiotherapy the clinical context in which the consequences of prior endocrine-driven adaptation may become most apparent.

The biologic interaction between radiotherapy and hormonal therapy in prostate cancer is already well established at the clinical level, where ADT improves outcomes when combined with radiation in appropriately selected patients. Mechanistically, this synergy is increasingly understood through the AR–DDR axis. AR regulates transcription of DNA repair genes, and suppression of AR signaling can impair repair of radiation-induced DNA double-strand breaks (5, 7, 8). Experimental studies have shown that castration or second-generation AR pathway inhibition can radiosensitize prostate cancer tissue and models by disrupting repair capacity (7, 8). These observations are typically interpreted as evidence of therapeutic synergy, but they also imply something broader: tumors that adapt to sustained endocrine pressure by restoring repair competence or by developing alternative repair-supportive programs may become relatively less sensitive to radiotherapy over time (5, 31).

Inflammatory signaling adds a second layer to this interaction. IL-6/STAT3 activation has been linked to prostate cancer radioresistance through upregulation of DDR-associated molecules including ATM, ATR, BRCA1, and BRCA2 (9). This suggests that inflammatory adaptation may, at least in part, offset the radiosensitizing effects initially achieved by AR suppression. In other words, while endocrine therapy may transiently weaken AR-dependent repair biology, a subsequent inflammatory program could help reconstruct a more survival-permissive and repair-supportive phenotype through alternative signaling routes (9, 19). This possibility is especially relevant in tumors exposed to prolonged endocrine pressure before or during radiotherapy, where adaptive inflammatory and stromal feedback may have time to reshape treatment response.

Radiotherapy also exerts important immunobiologic effects that may interact with this adaptive background. Beyond inducing DNA damage, RT can alter innate immune signaling, cytokine production, antigen presentation, and tumor–host communication (13, 14). Clinical proteomic and metabolomic studies in prostate cancer have shown that radiotherapy induces measurable innate immune responses (13), while broader radiobiologic analyses describe RT as an immunomodulatory intervention capable of generating both antitumor and protumor inflammatory consequences depending on context (14). From the perspective of the present hypothesis, this matters because tumors entering radiotherapy with pre-existing endocrine- and inflammation-associated reprogramming may respond differently to these RT-induced perturbations than tumors that remain more strongly AR-dependent and less plastic.

Epigenetic context is likely to be central to this interaction. Chromatin accessibility influences transcriptional responses to stress, the execution of DDR programs, and the maintenance of treatment-tolerant cell states (3, 31). In prostate cancer, emerging discussions of radioresistance increasingly place cell-state plasticity and chromatin regulation alongside classical DDR mechanisms (26, 30, 31). If endocrine therapy and inflammatory signaling have already reshaped the epigenetic landscape before radiation is delivered, then the tumor may encounter RT in a biologic state predisposed toward survival, repair competence, immune evasion, or lineage flexibility. Conversely, radiotherapy itself may further select for, and in some contexts reinforce, cells capable of mounting stress-adaptive transcriptional and inflammatory responses, raising the possibility of a feed-forward interaction between endocrine priming and RT-associated biologic stress (14, 30, 32). In this way, RT is not simply a passive endpoint affected by prior biology; it is a biologic challenge that can functionally reveal the consequences of prior adaptation.

This framework may help explain why tumors exposed to apparently similar endocrine–radiotherapy combinations still show heterogeneous responses. One plausible inference is that not all endocrine-exposed tumors evolve along the same adaptive trajectory. Some may remain predominantly AR-dependent and therefore relatively radiosensitizable, whereas others may transition toward inflammatory-high and epigenetically plastic states with reduced radiation responsiveness (30, 32). This interpretation does not replace established determinants of radioresponse such as intrinsic DDR capacity, hypoxia, clonal heterogeneity, or microenvironmental context. Rather, it proposes that endocrine-driven inflammatory–epigenetic adaptation may represent an underrecognized additional axis through which radiotherapy response becomes biologically stratified.

Taken together, radiotherapy is the clinically revealing context of the present model because it is where prior biologic adaptation becomes measurable through treatment response, local control, and biomarker-defined resistance trajectories. This supports more temporally informed biomarker strategies for endocrine-exposed disease (5, 9, 32).

### Actionable predictions and translational implications

**Figure 2:**
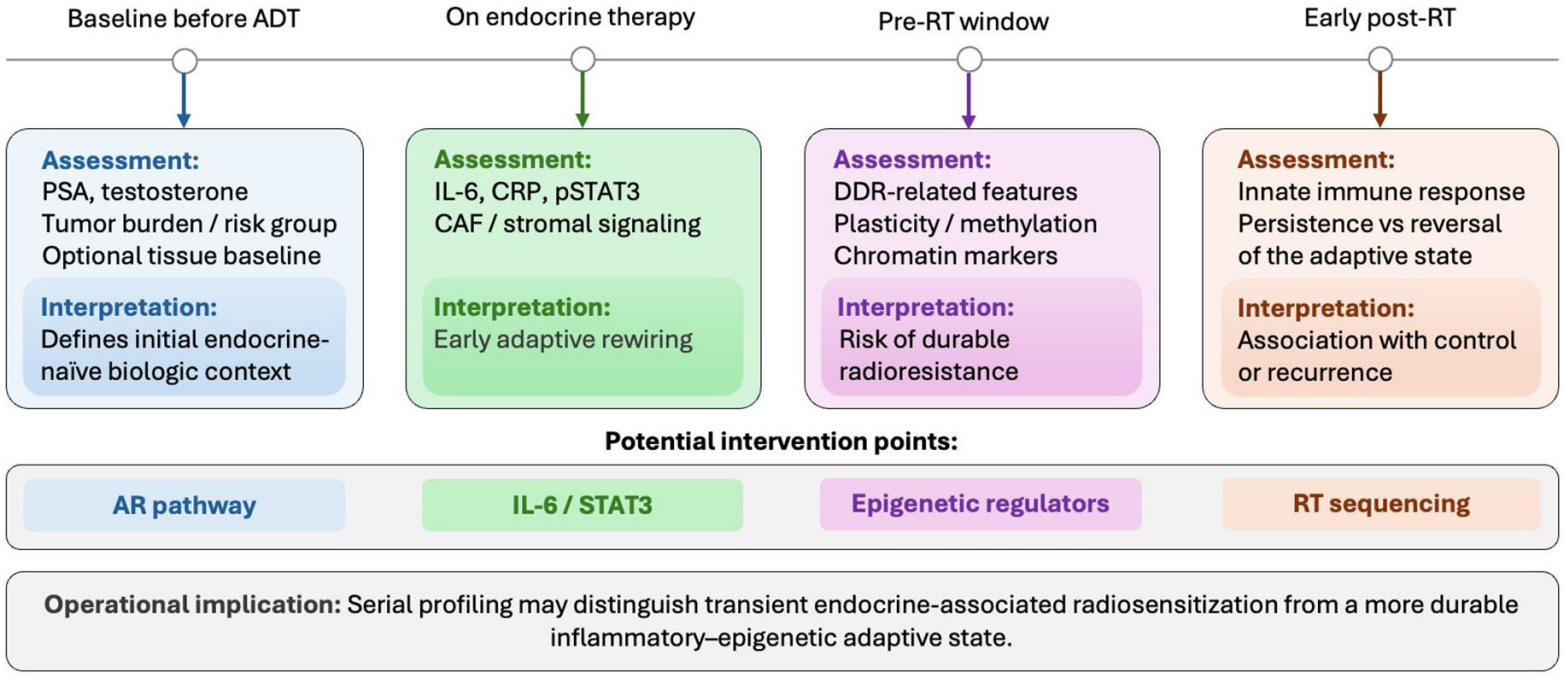
Translational roadmap for temporally resolved biomarker assessment in endocrine-exposed prostate cancer undergoing radiotherapy. The figure organizes the proposed translational strategy around four biologically relevant windows: baseline before androgen deprivation therapy (ADT), during endocrine exposure, the pre-radiotherapy (pre-RT) window, and the early post-radiotherapy phase. Within each window, candidate assessments are paired with their intended biologic or clinical interpretation to illustrate how serial profiling may distinguish transient endocrine-associated radiosensitization from a more durable inflammatory–epigenetic adaptive trajectory associated with relative radioresistance. Potential intervention points involving the AR pathway, IL-6/STAT3 signaling, epigenetic regulators, and radiotherapy sequencing are shown as secondary translational considerations. ADT, androgen deprivation therapy; AR, androgen receptor; CAF, cancer-associated fibroblast; CRP, C-reactive protein; DDR, DNA damage response; IL-6, interleukin-6; pSTAT3, phosphorylated signal transducer and activator of transcription 3; RT, radiotherapy.

If the proposed framework is valid, several testable predictions should follow across molecular, translational, and clinical levels. **Table 2** summarizes representative biomarker candidates, sampling windows, and validation approaches, while **Figure 2** highlights biologically relevant timepoints and potential intervention nodes across the treatment course. The central operational implication is that serial profiling should be more informative than baseline-only assessment for detecting emerging adaptive resistance.

**Table 2:** Testable predictions, candidate biomarkers, and validation strategies derived from the proposed endocrine-driven inflammatory–epigenetic model of prostate cancer radioresistance.

Prediction	Candidate biomarkers/features	Sample source	Suggested timing	Potential validation approach
A subset of endocrine-exposed tumors develops adaptive inflammatory activation	Serum IL-6, CRP, tissue pSTAT3, stromal inflammatory features, TIL density, CD8+/Treg ratio, TAM polarization features	Serum ± tissue	Before ADT and after defined endocrine exposure before RT	Prospective biomarker-embedded cohort with predefined serial serum collection and optional paired tissue analysis to identify emergence of inflammatory-high adaptive states with optional paired tissue immune-microenvironment analysis when available.
Inflammatory-high tumors show attenuated endocrine-associated radiosensitization	IL-6/STAT3 activity, DDR-related features, repair-supportive transcriptional programs	Serum ± tissue	Pre-RT window after endocrine exposure	Pre-RT correlative stratification study using circulating inflammatory profiling, with optional tissue analysis when available, to link adaptive inflammatory state to RT response, early PSA kinetics, and short-term biochemical or local control endpoints
Epigenetically plastic tumors evolve toward more durable adaptive radioresistant states	NSD2, EZH2, methylation signatures, lineage-plasticity markers, cfDNA-based epigenetic features	cfDNA ± tissue	During endocrine exposure, pre-RT, and early post-RT	Serial translational study using a circulating-first strategy based on cfDNA epigenetic profiling, with tissue analysis when available, to evaluate temporal state-transition dynamics and subsequent recurrence-pattern or control outcomes
RT-induced immune responses differ according to pre-existing endocrine–associated adaptive state	Innate immune response markers, cytokine dynamics, inflammatory response kinetics	Serum/plasma	Early post-RT	Paired pre-RT/post-RT biologic response study stratified by adaptive state to assess whether RT-induced inflammatory dynamics differ according to antecedent endocrine-associated reprogramming

The table summarizes the principal biologic and translational predictions arising from the present hypothesis, together with representative biomarker candidates, sample sources, suggested timing windows for assessment, and potential validation approaches. It is intended to provide a practical framework for prospective biomarker-embedded studies evaluating how endocrine exposure, inflammatory signaling, epigenetic plasticity, and radiotherapy response may interact over time. ADT, androgen deprivation therapy; cfDNA, cell-free DNA; CRP, C-reactive protein; DDR, DNA damage response; EZH2, enhancer of zeste homolog 2; IL-6, interleukin-6; pSTAT3, phosphorylated signal transducer and activator of transcription 3; RT, radiotherapy; TAM, tumor-associated macrophage; TIL, tumor-infiltrating lymphocyte; Treg, regulatory T cell.

First, endocrine therapy pressure should not only alter AR-dependent transcription, but also induce measurable adaptive inflammatory signaling in a subset of tumors exposed to sustained hormonal suppression, particularly within the IL-6/STAT3 axis (10, 19). Clinically, this implies that inflammatory biomarkers such as IL-6, CRP, and tissue-level pSTAT3 should be considered dynamic indicators of treatment adaptation rather than static prognostic markers alone. In the context of this hypothesis, the most informative question is not simply whether inflammatory signaling is present, but whether it emerges, persists, or intensifies during endocrine exposure before radiotherapy.

Second, tumors with persistent inflammatory activation under endocrine pressure should demonstrate features of attenuated endocrine-associated radiosensitization. The most direct mechanistic prediction is that IL-6/STAT3-high states will be associated with relative upregulation of DDR-supportive molecules or transcriptional programs that favor survival after radiation exposure (5, 9, 19). This is biologically plausible because IL-6 signaling has been linked to prostate cancer radioresistance through ATM, ATR, BRCA1, and BRCA2 regulation, while AR-linked DDR biology remains central to radiation response more broadly (5–9). In practical terms, this means that endocrine-treated tumors entering radiotherapy may not all behave equally: a subset may remain predominantly AR-dependent and therefore relatively radiosensitizable, whereas others may evolve toward a more inflammatory, repair-supportive, and less radiation-responsive state (30, 32).

Third, these adaptive states should exhibit epigenetic correlates of stabilization. If inflammatory signaling functions as the amplifier and epigenetic remodeling as the stabilizer of resistance, then inflammatory-high tumors should also show chromatin, methylation, or lineage-plasticity features consistent with more durable cell-state change (2, 12, 16, 24, 25). This prediction is especially important because it suggests that resistance biomarkers should not be restricted to serum cytokines or single-gene measures, but may require integrated assessment of inflammatory state, epigenetic plasticity, and radiogenomic context (12, 32).

From a translational perspective, the most immediate implication is the need for temporally resolved biomarker sampling rather than one-time baseline profiling. A practical framework could include assessment before initiation of endocrine therapy, a second sampling point during endocrine exposure and before radiotherapy, and an early post-radiotherapy sampling point. At minimum, such a panel could include serum IL-6 and CRP as inflammatory markers, testosterone or castrate-state verification as endocrine context markers, and selected tissue-based or circulating epigenetic features depending on feasibility (12, 19). If tissue is available through clinically indicated or protocol-justified sampling, additional exploratory endpoints could include pSTAT3, NSD2, EZH2, or methylation-linked indicators of lineage transition and state stabilization (16, 19, 27). When tissue is available, a practical and broadly accessible extension of this framework would be immunohistochemical assessment of the tumor immune microenvironment, including tumor-infiltrating lymphocyte (TIL) density, CD8+/Treg balance, and tumor-associated macrophage polarization, as these may help clarify whether adaptive inflammatory signaling is occurring primarily in a tumor-intrinsic, stromal, or immune-influenced state. Such markers would complement pSTAT3 and other inflammatory readouts without requiring specialized platforms and may improve biologic interpretation of tissue-based adaptive states (33, 34). However, repeated tissue sampling is unlikely to be feasible as a routine serial strategy in most radiotherapy settings. This type of serial design is more informative than baseline-only assessment because the present hypothesis concerns adaptive biologic change over time rather than fixed pretreatment state (**Figure 2**).

A practical next step would be a prospective biomarker-embedded study in endocrine-treated prostate cancer patients undergoing radiotherapy, with serial assessment at three predefined biologic windows: before ADT initiation, after defined endocrine exposure and immediately before RT, and early after RT. A suitable primary translational endpoint would be dynamic transition toward an adaptive inflammatory–epigenetic state, captured through change in serum markers such as IL-6 and CRP together with selected tissue-based or circulating features of epigenetic plasticity. Key clinical correlates could include early PSA response, biochemical control, local control, and recurrence pattern according to baseline and pre-RT adaptive state. Even such a relatively simple design could help determine whether biologic adaptation under endocrine pressure identifies tumors in which the initial radiosensitizing effect of AR suppression is later attenuated. A practical prototype study could distinguish three endpoint layers explicitly: feasibility endpoints (serial sampling success and assay robustness), biologic endpoints (dynamic transition toward an adaptive inflammatory–epigenetic state), and clinical correlates (early PSA kinetics, biochemical control, local control, and recurrence pattern according to baseline and pre-RT adaptive state).

The radiotherapy setting is particularly suitable for testing this framework because treatment response can be linked to defined clinical and molecular endpoints. At the clinical level, one could examine whether endocrine-exposed patients with inflammatory-high and/or epigenetically plastic signatures have inferior biochemical control, greater residual disease burden, or distinct recurrence patterns after radiotherapy. At the biologic level, one could ask whether RT-induced innate immune responses differ according to the pre-existing endocrine–inflammatory state of the tumor (13, 14). More broadly, prospective biomarker-embedded studies integrating inflammatory, epigenetic, and radiogenomic features may help distinguish tumors that remain endocrine-sensitive from those evolving toward adaptive radioresistant states (13, 32).

This model also has therapeutic implications, although these should be framed cautiously. If endocrine-driven inflammatory and epigenetic adaptation contributes to reduced radiotherapy sensitivity in selected tumors, then biomarker-informed combination strategies may become rational for biologically enriched high-risk states. One possibility is to evaluate radiotherapy and endocrine therapy together with selected modulation of inflammatory or epigenetic nodes in preclinical and early translational settings with particular attention to safety, overlapping toxicity, and appropriateness for curative-intent disease (4, 16, 19, 31). We are not suggesting immediate clinical adoption of unvalidated combinations. Rather, the present framework is intended to help prioritize which biologic subgroups and which adaptive pathways may be most informative to test first.

A further mechanistically relevant class of agents is PARP inhibitors. Because the present framework begins with AR-suppression-associated perturbation of DDR biology and later proposes adaptive restoration of repair-supportive competence in selected tumors, PARP inhibition is conceptually relevant, particularly in tumors harboring homologous recombination repair alterations or broader DDR vulnerability (35, 36). In this setting, one hypothesis is that PARP inhibition could help sustain or deepen radiosensitization in biologically selected subgroups, potentially counteracting the adaptive resistance trajectory proposed here. This possibility remains speculative in the present framework and should be viewed as a testable translational implication rather than a basis for immediate clinical incorporation into RT combinations.

Several specific hypotheses emerge from this framework. Patients who remain strongly AR-dependent and do not develop inflammatory amplification during endocrine exposure may derive greater and more sustained radiosensitization from combined ADT and RT than patients who transition toward inflammatory–epigenetic adaptation. Conversely, IL-6/STAT3 activation under endocrine pressure may identify tumors in which the radiosensitizing effect of hormonal therapy is transient and subsequently offset by repair-supportive inflammatory rewiring (5, 9, 19). Epigenetic plasticity markers may further help distinguish tumors that are merely endocrine-resistant from those actively evolving toward more durable, relatively radioresistant states (2, 16, 24, 25). These are not yet validated claims, but they are tractable and experimentally testable predictions.

Taken together, the translational strength of this hypothesis lies in its ability to organize future research around four practical questions: which tumors adapt, when adaptation occurs, how that adaptation becomes stabilized, and whether it predicts radiotherapy response. Rather than treating endocrine resistance, inflammatory signaling, epigenetic plasticity, and radioresistance as parallel literatures, the present framework supports prospective studies designed to capture their temporal and biologic interdependence. In that sense, its main value is not only explanatory, but operational: it provides a roadmap for biomarker design, mechanistic validation, and endocrine-aware personalization of radiotherapy in prostate cancer.

## Discussion

The central premise of this article is that, in endocrine-exposed prostate cancer, radioresistance may in some cases represent the downstream expression of an adaptive trajectory rather than solely a static baseline property. In this framework, endocrine therapy initially perturbs AR-dependent DDR biology and creates a radiosensitizing window, but in a subset of tumors sustained AR pathway pressure may subsequently promote inflammatory amplification and epigenetic stabilization of treatment-tolerant states that attenuate that benefit.

This model should be interpreted across three levels of evidence. First, several bilateral mechanistic links are well supported, including AR–DDR coupling, IL-6/STAT3-associated treatment adaptation and DDR-supportive signaling, and epigenetic stabilization of lineage plasticity and resistant cell states (2–10, 16, 17, 19, 23–25). Second, these domains are biologically compatible and plausibly cooperative under sustained endocrine pressure. Third, the full temporally ordered sequence proposed here remains inferential and hypothesis-generating. The conceptual advance therefore lies not in proposing a new pathway, but in reframing endocrine-associated radiosensitization as potentially dynamic over time and in positioning radiotherapy as the clinical context in which that divergence becomes clinically visible.

This framework positions radiotherapy not merely as an endpoint influenced by pre-existing biology, but as the clinically revealing context in which the consequences of prior adaptation become functionally apparent. Clinical synergy between radiotherapy and ADT is well established, and mechanistic studies have shown that AR suppression can radiosensitize prostate cancer by impairing repair of radiation-induced DNA damage (6–8). However, these same observations imply that tumors capable of adapting to sustained endocrine pressure through inflammatory rewiring and epigenetic stabilization may eventually regain, replace, or bypass repair-supportive states, thereby attenuating radiosensitization. The study by Chen et al. is particularly relevant in this regard, as it provides a mechanistic route through which IL-6 signaling may promote radioresistance via regulation of ATM, ATR, BRCA1, and BRCA2 (9). In this way, the present model expands the interpretation of combined-modality treatment: the benefit of endocrine therapy with radiotherapy may depend not only on initial AR suppression, but also on whether adaptive inflammatory–epigenetic programs emerge during the treatment course.

An additional implication of this framework is that radiotherapy may not only reveal prior endocrine-driven adaptation, but in some contexts may also reinforce it. RT induces DNA damage, innate immune perturbation, cytokine signaling, and stromal stress responses that may interact with pre-existing endocrine- and inflammation-associated reprogramming (13, 14, 30, 31). In biologically susceptible tumors, this raises the possibility of a feed-forward loop in which endocrine exposure primes adaptive inflammatory and epigenetic states, while subsequent RT-associated stress further amplifies or stabilizes those same programs, thereby contributing to relative radioresistance over time. In the present manuscript, this possibility should be regarded as biologically plausible and hypothesis-generating rather than established, but it strengthens the rationale for studying serial biomarker dynamics before, during, and after radiotherapy rather than viewing RT only as a passive endpoint.

Fractionation may be an important modifier of this interaction. Conventional fractionation, moderate hypofractionation, and SBRT differ in dose per fraction, overall treatment duration, and temporal spacing of biologic stress, and accumulating radiobiologic literature suggests that they may not engage tumor, stromal, inflammatory, and immune pathways identically (14, 37, 38). Reviews of RT–immune interactions indicate that hypofractionated and stereotactic regimens can produce distinct immunologic and microenvironmental effects compared with conventional fractionation, although these effects are context-dependent and not uniformly beneficial (37, 38). In prostate cancer, clinical immune-monitoring data after pelvic moderately hypofractionated RT also support the principle that fractionation and field design can shape systemic immune and cytokine responses (39). It is therefore biologically plausible that these regimens do not engage the proposed endocrine-driven inflammatory–epigenetic axis in identical ways. For example, longer conventional courses may provide more opportunity for cumulative adaptive signaling and stromal feedback, whereas larger-fraction regimens may impose different forms of acute stress selection and immune perturbation. At present, however, these possibilities remain insufficiently defined, and the present model should not be interpreted as assigning a definitive pro-adaptive or anti-adaptive effect to any particular fractionation strategy. Rather, fractionation should be considered a key experimental and translational variable in future studies testing this hypothesis.

The model also offers a biologically plausible explanation for heterogeneity of response among tumors exposed to apparently similar endocrine–radiotherapy combinations. One reasonable inference is that not all endocrine-exposed tumors follow the same adaptive trajectory. Some may remain predominantly AR-dependent and therefore relatively radiosensitizable, whereas others may transition toward inflammatory-high, epigenetically plastic, or lineage-divergent states with reduced radiation responsiveness (30, 32). This interpretation is supported indirectly by emerging data showing that endocrine therapy can actively reshape the landscape of accessible tumor states rather than simply select for a fixed resistant clone (2, 16, 18, 24, 25). If this is correct, then radioresistance may not always be a static pre-existing property of the tumor, but may in some cases emerge as a treatment-associated biologic state influenced by sequencing, duration of endocrine exposure, and adaptive microenvironmental feedback.

An important strength of the present framework is that it is mechanistically anchored and experimentally tractable. It does not rely on abstract endocrine–immune–epigenetic interaction as a conceptual possibility alone; rather, it builds on specific literature-supported connections. AR signaling regulates DNA repair genes (5–8). IL-6/STAT3 signaling contributes to progression, treatment adaptation, and DDR-supportive radioresistance-associated programs (9, 10, 19). Epigenetic regulators such as NSD2, EZH2, and methylation-associated remodeling support lineage plasticity and stabilization of resistant cell states (2, 12, 16, 17, 24, 25, 28, 29). Radiotherapy itself can induce measurable innate immune responses and reshape tumor–host signaling (13, 14). When considered together, these observations justify a model in which endocrine stress initiates adaptive change, inflammatory signaling amplifies it, and epigenetic remodeling stabilizes it in a form that becomes clinically meaningful in the radiotherapy setting.

At the same time, the limitations of this hypothesis should be stated clearly. The strongest evidence currently supports the bilateral relationships within the model rather than the fully integrated sequential loop. AR–DDR coupling is well established (5–8), IL-6/STAT3 is convincingly linked to progression and treatment adaptation (9, 10, 19), and epigenetic plasticity is increasingly implicated in endocrine resistance and lineage transition (2, 12, 16, 17, 23–25). By contrast, direct prospective evidence demonstrating that endocrine pressure induces inflammatory signaling that is subsequently epigenetically stabilized and clinically expressed as reduced radiotherapy sensitivity in patients remains limited. The present article therefore proposes a unifying and testable framework rather than claiming that the full sequence has already been proven. This distinction is essential, because the value of the model lies in its integrative and translational utility, not in overstating the current level of proof.

A second limitation is that radioresponse in prostate cancer is inherently multifactorial. Hypoxia, intrinsic DDR capacity, oxidative stress handling, cell-state composition, clonal heterogeneity, stromal context, dose and fractionation, and host immune factors all influence radiotherapy outcomes (14, 30, 31). The model proposed here should therefore be understood as one potentially important axis of adaptation rather than a universal explanation for all prostate cancer radioresistance. Likewise, IL-6/STAT3 is unlikely to be the only inflammatory pathway involved. Other cytokines, SASP-associated mediators, damage-associated molecular patterns, and tumor–microenvironment feedback loops may also contribute, particularly under repeated therapeutic stress (19–21). This should be interpreted not as a weakness of the framework, but as an indication that the present model likely captures a biologically central core that future studies can refine and expand.

A third limitation concerns clinical scope. Although the framework may have broader conceptual relevance across prostate cancer treatment states, it is likely to be most biologically informative in endocrine-exposed disease under sustained AR pathway pressure, particularly where sufficient time exists for adaptive inflammatory and epigenetic rewiring to emerge before or during radiotherapy.

A further practical issue is how to define the clinical circumstances in which this framework is most plausible. At present, the available evidence does not support a precise minimum duration of endocrine exposure required for adaptive inflammatory–epigenetic remodeling to occur, and we therefore avoid proposing a fixed temporal cutoff. In the present context, “sustained” endocrine exposure should be understood as biologically meaningful AR-pathway suppression extending beyond an acute radiosensitizing window and allowing sufficient time for adaptive transcriptional, inflammatory, stromal, and epigenetic rewiring to emerge (2, 5–10, 16, 18, 19, 23–25). This threshold is likely to be context-dependent rather than uniform across patients or treatment settings. The model may also vary according to endocrine modality and intensity. Classical castration-based approaches and more potent next-generation AR pathway inhibitors may not impose identical selective pressures, and deeper or more prolonged AR suppression may plausibly increase the opportunity for adaptive inflammatory and epigenetic state transitions, although this remains to be demonstrated prospectively (2, 5, 15, 16, 18, 23–25). Clinically, the framework is therefore most plausibly relevant in treatment scenarios such as high-risk localized or locally advanced disease treated with neoadjuvant/adjuvant ADT plus RT, and selected salvage settings in which meaningful endocrine exposure precedes or overlaps with radiotherapy. By contrast, its applicability is likely lower in low-risk disease, in definitive RT settings with little or no hormonal exposure, or where endocrine pressure is too limited in duration to permit biologically consequential adaptation. These distinctions should be viewed not as fixed exclusions, but as testable boundaries for future biomarker-embedded studies.

A fourth limitation concerns biomarkers and implementation. The translational appeal of the model lies partly in its capacity to guide biomarker development, yet the field still lacks validated composite signatures that simultaneously capture endocrine state, inflammatory adaptation, epigenetic plasticity, and radiotherapy response. Serum IL-6 is measurable and biologically relevant, but by itself it is unlikely to be sufficiently specific. Tissue-based markers such as pSTAT3, NSD2, EZH2, methylation signatures, or lineage-plasticity-associated features may be more informative, but their clinical use will require harmonized sampling strategies, assay reproducibility, and prospective correlation with outcome (12, 16, 19, 25, 28, 32). Accordingly, the model should motivate prospective biomarker programs rather than premature claims of immediate clinical readiness.

A related practical limitation is biomarker feasibility. Although tissue-based markers such as pSTAT3, NSD2, EZH2, methylation signatures, and lineage-plasticity-associated features are mechanistically attractive, repeated prostate biopsies across endocrine exposure and the pre-radiotherapy window are ethically and logistically challenging and are unlikely to be feasible as a routine serial strategy in many patients (40). In this context, repeat tissue sampling should be viewed primarily as an exploratory option for selected prospective protocols, opportunistic translational substudies, or settings in which clinically indicated tissue is already available, rather than as a universally scalable biomarker approach. Likewise, serum IL-6 and CRP are measurable and biologically relevant, but their specificity is limited because both can be influenced by infection, chronic inflammatory conditions, treatment-unrelated host factors, and comorbidity (41). For this reason, they are better interpreted as longitudinal components of a broader composite biomarker framework than as standalone indicators of adaptive radioresistance. These constraints increase the appeal of less invasive strategies, including circulating tumor DNA methylation profiling, other blood-based epigenetic approaches, and extracellular vesicle or exosomal markers, which may offer a more feasible route for serial assessment of adaptive inflammatory–epigenetic state transitions (42, 43). At present, however, these assays remain investigational in this setting and require analytical standardization, clinical validation, and prospective correlation with outcome before they can support treatment decision-making.

A further limitation is that some of the most direct evidence for epigenetically stabilized lineage-divergent states derives from advanced or metastatic disease. Accordingly, extrapolation to earlier radiotherapy-treated settings should be viewed as biologically plausible rather than established, and may reflect partial adaptive programs rather than fully evolved advanced-disease phenotypes.

These limitations define the most important future research priorities. The first is temporal validation: studies should examine how inflammatory markers, AR-related repair programs, and epigenetic states change across biologically meaningful windows, including before endocrine therapy, after defined endocrine exposure and before radiotherapy, and early after radiotherapy. The second is state-specific validation, asking whether inflammatory-high and epigenetically plastic tumors indeed show reduced radiosensitivity or distinct recurrence patterns. The third is mechanistic intervention, testing whether selected inhibition of IL-6/STAT3 or epigenetic regulators can restore radiosensitivity in endocrine-adapted models. The fourth is integrated biomarker design, combining serum, tissue, and circulating nucleic acid approaches to distinguish tumors that remain endocrine-sensitive from those evolving toward inflammatory–epigenetic radioresistant states (2, 9, 12, 16, 19, 25, 32). These are realistic and testable next steps that align well with prospective translational frameworks in prostate cancer.

The clinical implications of the model also merit emphasis, albeit cautiously. The current use of ADT with radiotherapy is supported by strong clinical evidence, and nothing in this hypothesis argues against established standard-of-care combinations. Rather, the present framework suggests that the biology of endocrine exposure may matter as much as the presence or absence of endocrine therapy itself. Duration, timing relative to radiotherapy, intensity of AR suppression, and pre-existing tumor plasticity may all influence whether endocrine therapy remains radiosensitizing or whether compensatory inflammatory–epigenetic adaptation begins to attenuate that effect. If validated, this would have implications for patient stratification, biomarker-guided sequencing, and biologically enriched combination strategies incorporating inflammatory or epigenetic modulation (4, 16, 19, 29). At present, however, these implications should be regarded as research-generating rather than practice-changing.

A related issue is how baseline disease characteristics may contextualize the proposed trajectory. Tumor stage, ISUP grade group, and baseline PSA are clinically relevant because they influence disease burden, biologic aggressiveness, and the likelihood that patients receive prolonged and/or intensified endocrine exposure before radiotherapy (4, 5, 44). In that sense, these variables are not framed here as direct mechanistic drivers of the adaptive axis, but as practical modifiers of the clinical context in which the axis is most likely to emerge. The model is therefore most plausibly relevant in biologically higher-risk subgroups, including high-risk localized, locally advanced, and selected salvage settings, where sustained AR-axis suppression is more likely to precede or overlap with RT (44). By contrast, its applicability is likely lower in earlier-stage or lower-risk disease where endocrine exposure is absent, brief, or biologically less dominant. Future biomarker-embedded studies should therefore stratify analyses by disease stage, grade group, and baseline PSA to determine whether these variables influence the timing and magnitude of the proposed inflammatory–epigenetic shift.

A further caution concerns safety. Although biomarker-informed modulation of inflammatory or epigenetic nodes may be biologically attractive as a future strategy for selected endocrine-adapted tumors, such combinations cannot currently be regarded as ready for clinical adoption in curative-intent prostate radiotherapy. IL-6/STAT3-directed approaches may carry risks of immune perturbation or immunosuppression, while epigenetic agents may introduce overlapping hematologic and systemic toxicities that could be particularly relevant when combined with pelvic radiotherapy and androgen deprivation therapy (45–47). In a setting where standard combined-modality treatment is already potentially curative, any intensification strategy would require an especially high threshold of biologic justification, safety characterization, and patient selection. Accordingly, the present framework should be interpreted as supporting preclinical and early translational exploration of these concepts, not immediate therapeutic incorporation into routine clinical care.

Overall, the value of this framework lies in its ability to organize future work around a clinically relevant question: why some endocrine-exposed tumors appear to remain radiosensitizable whereas others may evolve toward adaptive states associated with relative radiotherapy resistance. The individual biologic links underlying the model are supported by substantial evidence, but their full temporal and clinical integration remains to be tested. This makes endocrine-driven inflammatory–epigenetic adaptation less a settled mechanism than a focused translational hypothesis that can now be examined through prospective biomarker, experimental, and treatment-sequencing studies.

## Conclusion

Prostate cancer radioresistance is often interpreted through separate frameworks centered on AR signaling, DDR efficiency, inflammatory adaptation, or lineage plasticity. We propose a more integrated and temporally specific view for endocrine-exposed disease: endocrine therapy may initially radiosensitize tumors through AR–DDR perturbation, yet in a subset of tumors sustained endocrine pressure may subsequently promote inflammatory amplification and epigenetic stabilization of adaptive states that attenuate that benefit. The value of this model lies not in claiming that the full sequence has already been proven, but in offering a biologically coherent and testable explanation for why endocrine-exposed tumors may diverge in radiotherapy responsiveness over time. If validated, this framework could support temporally resolved biomarker development and more biologically informed endocrine-aware radiotherapy stratification, particularly in treatment settings involving sustained endocrine exposure before or during RT.

## Data Availability

The original contributions presented in the study are included in the article. Further inquiries can be directed to the corresponding author.
